# Association between Serum Urate and Risk of Hypertension in Menopausal Women with XDH Gene

**DOI:** 10.3390/jcm8050738

**Published:** 2019-05-23

**Authors:** Jong-Han Lee, Tae Hwa Go, San-Hui Lee, Juwon Kim, Ji Hye Huh, Jang Young Kim, Dae Ryong Kang, Seongmun Jeong, Sang-Baek Koh, Jung Ran Choi

**Affiliations:** 1 Department of Laboratory Medicine, Yonsei University Wonju College of Medicine, Wonju 26426, Korea; cello425@yonsei.ac.kr (J.-H.L.); juwon76@yonsei.ac.kr (J.K.); 2 Center of Biomedical Data Science, Institute of Genomic Cohort, Yonsei University Wonju College of Medicine, Wonju 26426, Korea; thgo1123@gmail.com (T.H.G.); dr.kang@yonsei.ac.kr (D.R.K.); 3 Department of Obstetrics and Gynecology, Yonsei University Wonju College of Medicine, Wonju 26426, Korea; sanaram@yonsei.ac.kr; 4 Department of Internal Medicine, Yonsei University Wonju College of Medicine, Wonju 26426, Korea; png1212@yonsei.ac.kr (J.H.H.); kimjang713@gmail.com (J.Y.K.); 5 Genome Editing Research Center, Korea Research Institute of Bioscience and Biotechnology, Daejeon 34141, Korea; likemun@gmail.com; 6 Department of Preventive Medicine and Institute of Occupational and Environmental Medicine, Wonju College of Medicine, Yonsei University, Wonju 26426, Korea; kohhj@yonsei.ac.kr; 7 Institute of Genomic Cohort, Yonsei University Wonju College of Medicine, Wonju 26426, Korea

**Keywords:** serum urate, menopause, hypertension, xanthine dehydrogenase, cross-sectional cohort study

## Abstract

Elevated serum urate (sUA) concentrations have been associated with an increased risk of hypertension. We aimed to examine the association of sUA concentration on the risk of hypertension in pre- and post-menopausal women and investigated the association between the polymorphism of the xanthine dehydrogenase gene and the risk of hypertension. Among 7294 women, 1415 premenopausal and 5879 postmenopausal women were recruited. Anthropometric parameters as risk factors of hypertension were identify by logistic regression models. In addition, we investigated an association between xanthine dehydrogenase gene and sUA and their combined associations on the risk of hypertension. Body mass index (BMI) and waist circumference (WC) were significantly increased in accordance to the increase of sUA levels (*p* < 0.001). Multivariate logistic regression analysis showed postmenopausal women with a high sUA and high BMI were 3.18 times more likely to have hypertension than in those with normal and lower sUA (Odds ratio: 3.18, 95% confidence interval: 2.54–3.96). Postmenopausal women with a high WC were 1.62 times more likely to have hypertension than in those with normal and lower sUA. Subjects with the AG genotype of rs206860 was found to be at lower risk of hypertension (odd ratio: 0.287, 95% confidence interval: 0.091–0.905, *p* = 0.033). This cross-sectional study indicated a high sUA is associated with a higher risk of hypertension in postmenopausal women. Further well-designed prospective studies in other populations are warranted to validate our results.

## 1. Introduction

An elevated serum urate (sUA) concentration is a common phenomenon in subjects with hypertension, insulin resistance, or obesity [1], and previous epidemiological studies have demonstrated that high sUA concentrations are associated with an increased risk of hypertension [1,2,3,4,5]. Although longitudinal studies showed sUA might play a role in the development of hypertension, the association between sUA and blood pressure may be affected by various factors [5,6,7]. However, it is not clear whether urate elevation is the cause or a consequence of hypertension [1,6,8].

Urate is the catabolic end-product of endogenous and dietary purine metabolism in human, and is mainly produced by xanthine oxidase, which is involved in the production of reactive-oxygen species (ROS) [5,9,10,11]. In addition, excessive sUA accumulation can cause various diseases [11,12]. Several studies using animal models and cell cultures have identified mechanisms whereby high sUA concentrations might lead to hypertension by reducing endothelial nitric oxide release and activating the renin–angiotensin system leading to smooth muscle cell proliferation [13,14,15,16]. Furthermore, associations between sUA and metabolic disorders are gender dependent. sUA concentrations tend to be lower in women than in men, partially due to the uricosuric effect of estrogens [10]. In addition, sUA concentrations seem to be increased in both physiologic and post-surgical menopause independently of other confounders [9,17], presumably due to the uricosuric effect of estrogens and hormone replacement therapy induced sUA reduction [18]. Estrogens have some indirect uricosuric effects that can contribute to modulate the urate before and after menopause. The sUA concentration is unlikely to reflect urate production, as an increased sUA production is compensated by increased excretion to maintain sUA within the normal range [19]. To the best of our knowledge, urate production has a limited influence on sUA concentration and both, urate concentration and the production, might influence blood pressure by a different underlying mechanism.

Mammalian xanthine oxidoreductase (XOR) has the characteristics of being found in two interconvertible forms: constitutively expressed in vivo NAD+-dependent xanthine dehydrogenase (XDH, EC 1.1.1.204) and post-transcriptionally modified xanthine oxidase (XO, EC 1.1.3.22) [20]. The XOR enzyme exists in XDH form, but when released into the circulation it is converted into XO. Although XDH preferentially reduces NAD+, both forms of the enzyme can also reduce molecular oxygen to form the ROS superoxide and hydrogen peroxide. Wu et al. reported the *XDH* gene might be associated with constitutional susceptibility to hypertension [21]. XDH alters xanthine oxidase by reversible sulfhydryl oxidation or by irreversible proteolytic modification, and the production of urate results from the metabolism of purines by XDH [21,22].

We examined the association between sUA and hypertension risk in post-menopausal women, and investigated the association between the polymorphism of *XDH* gene and the risk of hypertension.

## 2. Methods

### 2.1. Study Population

This study was conducted with participants from a population-based cohort within the Korean Genome and Epidemiology Study on Atherosclerosis Risk of Rural Areas in the Korean General Population (KoGES-ARIRANG) to assess the genetic and environmental etiology of common metabolic common metabolic and cardiovascular diseases in South Koreans [23,24]. The KoGES-ARIRANG cohort study contained all adults aged 40–70 years that resided in rural areas of Wonju and Pyeongchang, Gangwon-do, Republic of Korea.

**Figure 1:**
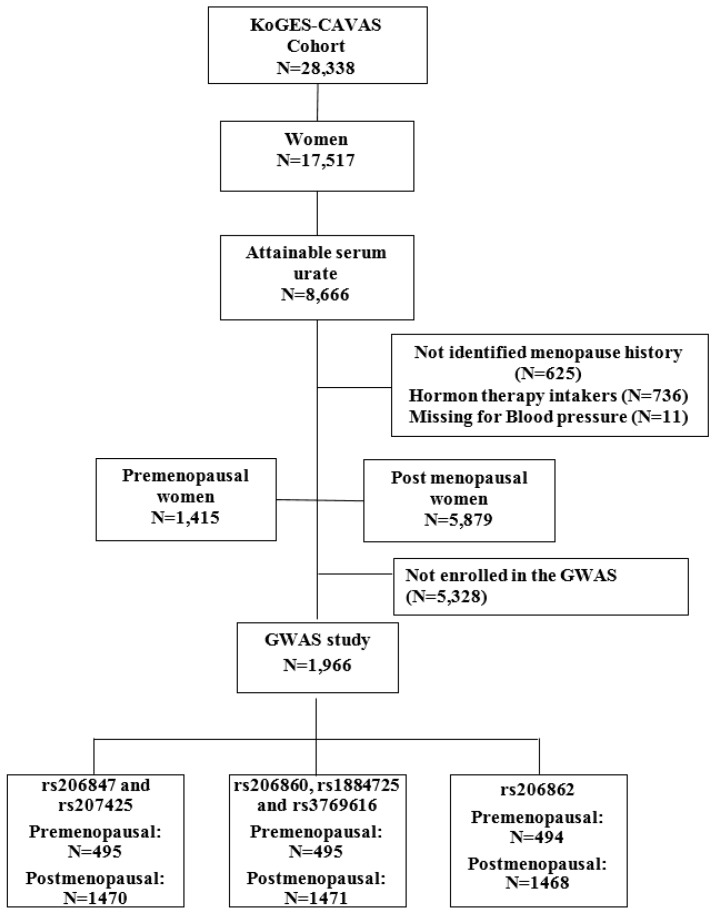
Flow chart of study populations in the KoGES-CAVAS cohort. Abbreviations: KoGES-CAVAS, Korean Genome and Epidemiology Study on Atherosclerosis Risk of Rural Areas in the Korean General Population; GWAS, genome wide association study.

In this cross-sectional study, the baseline survey was performed from November 2005 to January 2008 and contained 28,338 adults aged 40 to 70 years. Among 17,517 women, 8666 women with a sUA level were included in this study. After excluding 736 with experience of hormone therapy, 625 with no history of menopause, and 11 missing for blood pressure, a total of 1415 premenopausal and 5879 postmenopausal women comprised in this cross-sectional study (Figure 1). Subjects who were treated antihypertensive drugs were 1991 participants. Those who experienced anti-hypertensive drugs were considered having hypertension. Additionally, we excluded the participants without genotype, 3666 participants with the genetic variations in *XDH* were eligible for this cross-sectional study. The study protocol was approved by the Institutional Review Board of Wonju Severance Christian Hospital.

### 2.2. Data Collection

At baseline examination, study participants completed a standardized medical history and lifestyle questionnaire and underwent a comprehensive health examination according to standard procedures.

Body weight and height were measured, while participants were wearing light indoor clothing without shoes. Waist circumference (WC) was measured in a horizontal plane, midway between the inferior margin of the ribs and the superior border of the iliac crest using a tape measure (SECA-200, SECA, Hamburg, Germany).

Systolic (SBP) and diastolic blood pressures (DBP) were measured twice in right arms within 5 min using a standard mercury sphygmomanometer (Baumanometer, Copiague, NY, USA). The means of the two blood pressure readings were used for the data analyses. Hypertension was defined as a SBP of ≥140 mmHg or a DBP ≥90 mmHg and/or current treatment with antihypertensive medications at the baseline survey. All the participants were examined after fasting.

A venous blood sample was drawn from study participants after fasting for >12 h or overnight. Serum aliquots were stored at −80 °C until thawed for analysis. The sUA was measured by an enzymatic coloric method that can detect the absorbance differences using uricase and peroxidase as reaction enzymes. Fasting glucose was measured using the hexokinase method. The serum concentrations of high-density lipoprotein (HDL) cholesterol, total cholesterol (TC), and triglycerides (TG) were determined using the enzymatic calorimetric method.

Alcohol and smoking habits were estimated using self-questionnaires. Individuals who had smoked ≥100 cigarettes in their lifetime were defined as current smokers, and those who had not smoked for ≥3 months were defined as ex-smokers. An interview was performed to confirm the use of medications for hypertension, and the status of regular physical exercise.

At baseline examination, study participants completed a standardized medical history and lifestyle questionnaire and underwent a comprehensive health examination according to standard procedures. At first, we estimated menopausal status using self-questionnaire. Women who had pre- and postmenopausal status were defined as “Have you always had your periods at regular 28-day intervals? Or, have you had not your periods for three months recently?” Also, an interview was performed to confirm the use of medications for hormone therapy.

### 2.3. Genome Wide Association Study Genotyping

Samples were analyzed using an Affymetrix Genome-Wide Human single nucleotide polymorphism (SNP) array 6.0, which contains 906,600 genome-wide SNPs and 946,000 copy number variations. Briefly, the genomic DNA was digested with two restriction enzymes (NSP I and Sty I) and processed according to the Affymetrix protocol. Digested segments were ligated to enzyme specific adaptors incorporating a universal PCR priming sequence. PCR amplification was performed using universal primers in a reaction optimized for the amplification of fragments between 200–1100 base pairs. A fragmentation step was then used to reduce the PCR products to segments of approximately 25–50 bp, which were then end-labeled using biotinylated nucleotides. The labeled products were then hybridized to a chip, washed, and detected. Images were analyzed using GeneChip Operating System software (Affymetrix, Santa Clara, CA, USA). Internal quality control measures were to ensure data fidelity, that is, a QC call rate (Dynamic Model algorithm) always was over 86% and correct identification of subject gender based on heterozygosity on the X chromosome. Genotype calling was performed using the Birdseed v2 algorithm [25].

### 2.4. Analysis of the XDH Genomic Polymorphism

For this study, *XDH* fragments were independently amplified by polymerase chain reaction (PCR). PCR products were purified and then sequenced using a BigDye Terminator v3.1 Cycle Sequencing Kit (Applied Biosystems, Foster City, CA, USA) and an ABI 3730 × 1 automated sequencer (Applied Biosystems). SNPs identified in the *XDH* gene by whole gene sequencing were genotyped. Genomic DNA was extracted from 5 mL of peripheral venous blood using a commercially available isolation kit (QuickGene SP Kit DNA whole blood, Fujifilm, Tokyo, Japan). Genotyping was performed using the TaqMan fluorogenic 5’ nuclease assay (Applied Biosystems) [25].

### 2.5. Statistical Analysis

We analyzed the study population divided in quartiles of sUA. Categorical variables were analyzed using the chi-square test and continuous variables were analyzed by ANOVA and post hoc using Scheffe’s test in pre- and post-menopausal women. Interactions between drinking status, body mass index (BMI), WC, TG, and sUA on hypertension were investigated. In order to identify an association between sUA and hypertension we analyzed multivariate logistic regression which was used to evaluate the independence of associations between sUA and risk of hypertension according to menopausal status after adjusting for fasting glucose, BMI, and WC. Additionally, there might be collinearity between BMI and WC. Consequently, we demonstrated a part of odds ratio for sUA and hypertension without considering all confounding factors including age. We used SBP as confounding factors because of one of components defined participants with hypertension and without hypertension. In additional analysis, we adjusted for age to investigate an association of sUA and clinical variables, because postmenopausal women were older than premenopausal women.

Results were expressed as ORs ratios and 95% confidence intervals (CI). All analyses were performed using SAS version 9.3 (SAS Institute, Cary, NC, USA), and SPSS version 23.0 (IBM Corp., Armonk, NY, USA). P value less than 0.05 was considered as statistically significant.

## 3. Results

### 3.1. Baseline Characteristics

The percentages of BMI, SBP, fasting glucose, and TG were significantly higher, and HDL cholesterol were significantly lower in the highest quartile of sUA (UA ≥ 5.0 mg/dL) (Table 1). BMI and WC were significantly higher in the participants with the highest quartile of sUA than those with the lowest quartile of sUA. (25.3 ± 3.4 vs. 23.6 ± 3.2; 85.2 ± 8.9 vs. 81.0 ± 9.0; respectively, *p* < 0.01) (Table 1). In the beginning of study design, we analyzed an association of sUA and hypertension with substantial confounding factors that related with sUA such as BMI and alcohol consumption. However, we did not confirm an association between sUA and hypertension after adjusted for all potential confounding factors.

### 3.2. Anthropometric Characteristics of Premenopausal and Postmenopausal Women with Hypertension

In postmenopausal women, the mean sUA values were higher in the participants in whom hypertension development was observed than in those who did not develop hypertension (4.72 ± 1.25 vs. 4.35 ± 1.03, *p* < 0.001). In the premenopausal women who developed hypertension, the baseline SBP and TC were 136.00 ± 16.44 mg/dL and 197.40 ± 33.90 mg/dL, respectively, and these parameters were higher than in those who did not develop hypertension (*p* < 0.001 vs. *p* = 0.002). There were no differences in smoking habits and the regular exercise status, between the pre- and postmenopausal women (Table 2).

### 3.3. Univariate and Multivariate Logistic Regression Analyses

In univariate logistic regression analysis, we investigated that postmenopausal women with a high sUA and high BMI were 3.13 times more likely to have hypertension than those with a normal BMI and a lower sUA (OR 3.13, 95% CI 2.67–3.66 vs. OR 1.85, 95% CI 1.63–2.11, respectively) (Table 3). A higher sUA level and TG were positively and significantly associated with the development of hypertension in postmenopausal women (OR 2.63, 95% CI 2.25–3.08) (Table 3). Pre-menopausal women with high sUA and high BMI showed 5.20 times more likely to have hypertension than those with normal BMI and a lower sUA (OR 5.20, 95% CI 3.48–7.78) (Table 3).

Multivariate logistic analysis after adjusting for age, SBP and BMI showed that a higher sUA and a high WC was found to be significantly associated with a 1.62-fold increased risk of hypertension in postmenopausal women (Table 3). After adjusting for age, SBP and BMI showed that a high sUA and a high TG were found to be significantly associated with a 2.08-fold increased risk of hypertension in postmenopausal women (OR 2.08, 95% CI 1.72–2.53) (Table 3).

### 3.4. Hypertension Risk of Participants according to Genotypes of Xanthine Dehydrogenase (XDH)

We found that participants with *XDH* rs206847 CC genotype had a risk of hypertension with statistical significance (OR=3.63). Multivariate logistic regression analysis showed a significantly lower risk of hypertension in women with rs206860AG genotypes than those with the wild type (AA) (OR = 0.26, 95% CI 0.08–0.89, *p* = 0.03) (Table 4). After adjusting for age, smoking status, alcohol consumption, and regular exercise, rs206826 AC genotype was associated with a decreased risk of hypertension (OR = 0.28 95 % CI 0.08–0.98). However, women with rs206847 CC and rs207425 GA genotypes had an elevated risk of hypertension.

## 4. Discussion

In the present study, an elevated sUA was observed to be positively associated with an increased risk of hypertension and postmenopausal women with high sUA and BMI were 3.13 times more likely to have hypertension than those who had low sUA and BMI. Furthermore, *XDH* rs206860 AG genotype was found to be associated with the decreased risk of hypertension.

The present study showed that hyperuricemia was significantly associated with elevated risk of hypertension after adjusting for known confounders. Several potential mechanisms might explain the association between sUA and hypertension [5,26]. The first involves insulin resistance. Elevated insulin levels cause low urinary ammonium levels and predispose the precipitation of sUA [5,26]. Furthermore, insulin resistance is known to contribute to the developments of several metabolic disorders that influence the development of coronary artery disease [5,27], and to increase postmenopausal sUA concentrations. The second involves the detrimental effect of an elevated sUA concentration on renal function. Hyperuricemia leads to hypertension and renal injury via a crystal-independent mechanism by stimulating the renin–angiotensin system and inhibiting neuronal nitric oxide synthase [5,28]. Also, as sUA in rats induces sodium excretion to decrease by the epithelial sodium channel, it therefore contributes hypertension [29]. The third mechanism involves endothelial dysfunction. Experimental evidence suggests a potentially causal role for urate in the pathogenesis of hypertension and atherosclerosis [28]. It was reported that sUA concentrations are higher in postmenopausal than in premenopausal women [30]. As far as we know, in postmenopausal women who did not receive estrogen hormone therapy, sUA concentration tended to increase due to a shortage of the uricosuric effect of estrogen.

Although the association between sUA and hypertension has been already demonstrated in epidemiological and clinical studies, the nature of the interaction between sUA and hypertension remains debatable. The sUA might not be an independent risk factor of hypertension after controlling for other risk factors, but some studies have reported sUA is predictive of hypertension and renal disease development after controlling for associated risk factors. However, a recent meta-analysis including 25 studies of 97,824 participants has shown that high sUA significantly predicts systemic hypertension [16].

In fact, urate found to have several beneficial and potentially detrimental biologic effects [31]. Our results suggest hyperuricemia dose-dependently predicts higher risks of hypertension as proportions of subjects with hypertension increased significantly with sUA quartile, which is consistent with the findings of previous studies. Interestingly, we found genetic variations of the rs206860 polymorphism of *XDH* gene might lower the risk of hypertension. To the best of our knowledge, the association between polymorphism of rs206860 and hypertension was not widely reported. rs206860 was investigated in the studies of advanced liver disease [32] and anti-tuberculosis drug-induced hepatotoxicity [33]. As far as we know, other SNPs were not directly reported regarding association between heterogeneity of SNPs and hypertension.

Other SNPs of *XDH* gene associated with hypertension were also reported. Recently, Scheepers et al. reported that mean arterial pressure and DBP increased approximately 1 mmHg less in carriers of minor alleles of *XDH* rs2043013 in a European population [19]. Yang J et al. reported multivariate logistic regression analysis showed a significant association between the three SNPs of *XDH* at rs2043013 and hypertension in men: 47686C>T and 69901A>C in the recessive model, and 67873A>C (N1109T) in the dominant model [34]. Wu B et al. showed the *XDH* gene polymorphisms rs1042039, rs1054889, and rs2073316 might be associated with hypertension in the rural Han Chinese population [21].

The present study has some limitations that warrant considerations. First, subjects were not analyzed over a follow-up period, and therefore, we could not individually evaluate whether the association between sUA concentration and new-onset hypertension was relevant over a longer period. Second, the genome wide association study (GWAS) population included a smaller number of postmenopausal women with hypertension than our basic subjects for each parameter analysis. Third, our findings might be differently applied to other populations, especially younger age groups of different ethnicities. As far as we know, *XDH* rs206860 and its associations with hypertension have not been studied. rs206860 of *XDH* is unknown genetic variant so far and further studies in gene discovery and function needs to be verified. Also, cross-sectional nature of study has limited power to make conclusions about causality.

In order to investigate an association between genetic variants in *XDH* and risk of hypertension, we examined confounding factors in multivariate logistic regression analysis gradually. We identified the optimal model adjusted for age, smoking status, alcohol consumption, regular exercise, systolic blood pressure, total cholesterol, and baseline body mass index. However, we recognized that women with hypertension were few and have very wide confidence intervals because some women do not have the genotype of *XDH*. Also, it is not possible to identify whether high urate leads to hypertension or not from a cross-sectional study

However, this study showed a significant association between sUA and hypertension risk in postmenopausal women. sUA might be a useful marker to predict disease modality and progression of chronic metabolic diseases such as hypertension in clinical practice.

## 5. Conclusions

This study suggests that sUA concentrations might be associated with an increased risk of hypertension in postmenopausal women and that the rs206860 polymorphism of the *XDH* gene might be associated with a low risk of hypertension in Koreans. It seems that sUA may be a cost-effective, applicable parameter to evaluate hypertension risk of postmenopausal women. Further well-designed, large-scale studies in other populations are warranted to validate our results.

## Figures and Tables

**Table 1:** Sociodemographic characteristics of the study population.

Variable	Serum Urate (sUA)				*p*-Value	Post-Hoc *
	Quartile 1 (<3.7 mg/dL)	Quartile 2 (3.7 ≤ 4.3 mg/dL)	Quartile 3 (4.3 ≤ 5.0 mg/dL)	Quartile 4 (≥5.0 mg/dL)		
Women						
Premenopausal	411 (24.3)	385 (23.4)	381 (20.0)	238 (11.6)	<0.01	
Postmenopausal	1282 (75.7)	1258 (76.6)	1524 (80.0)	1815 (88.4)		
Age	60.5 ± 10.6	60.2 ± 10.5	60.4 ± 10.3	62.6 ± 9.6	<0.01	a, b, c < d
Smoking status						
Non, ex-smoker	1039 (96.9)	978 (96.4)	1140 (96.9)	1116 (35.1)	0.2	
Current smoker	22 (3.1)	36 (3.6)	37 (3.1)	58 (4.9)		
Alcohol						
Yes	458 (27.1)	447 (27.3)	573 (30.1)	602 (29.3)	0.11	
No	1235 (72.9)	1193 (72.7)	1330 (69.9)	1451 (70.7)		
Weight (kg)	54.6 ± 8.5	56.1 ± 8.2	57.4 ± 8.7	58.8 ± 9.5	<0.01	a < b < c < d
Height (cm)	152.0 ± 6.2	152.6 ± 6.2	152.5 ± 5.9	152.1 ± 6.0	0.02	
BMI (kg/$m^{2}$)	23.6 ± 3.2	24.1 ± 3.0	24.7 ± 3.2	25.3 ± 3.4	<0.01	a < b < c < d
WC (cm)	81.0 ± 9.0	81.9 ± 8.8	83.4 ± 9.0	85.2 ± 8.9	<0.01	a < b < c < d
HC (cm)	91.7 ± 6.3	92.5 ± 6.2	93.3 ± 6.8	94.0 ± 6.9	<0.01	a < b < c < d
Creatinine (mg/dL)	0.8 ± 0.1	0.8 ± 0.1	0.9 ± 0.1	0.9 ± 0.3	<0.01	a < b < c < d
HDL-C (mg/dL)	47.9 ± 11.2	46.9 ± 10.9	45.8 ± 11.2	43.7 ± 10.4	<0.01	a, b > c > d
Fasting glucose (mg/dL)	97.9 ± 24.5	95.9 ± 19.3	98.0 ± 19.2	100.4 ± 21.1	<0.01	a < d, b < c < d
Total cholesterol (mg/dL)	199.0 ± 36.0	200.6 ± 34.7	203.7 ± 36.2	208.8 ± 38.6	<0.01	a < c < d, b < d
Triglycerides (mg/dL)	126.4 ± 67.2	130.7 ± 72.2	146.1 ± 83.4	171.8 ± 104.0	<0.01	a, b < c < d
Total protein (g/dL)	7.3 ± 0.4	7.3 ± 0.4	7.4 ± 0.4	7.5 ± 0.4	<0.01	a < c < d, b < d
Albumin (g/dL)	4.4 ± 0.2	4.4 ± 0.2	4.5 ± 0.2	4.5 ± 0.2	<0.01	a, b < c, d
GGT (I/U)	17.2 ± 15.7	17.8 ± 21.7	20.4 ± 28.2	24.8 ± 30.8	<0.01	a, b < c < d
SBP (mmHg)	122.3 ± 18.1	122.3 ± 17.7	123.6 ± 18.4	126.4 ± 18.0	<0.01	a, b, c < d
DBP (mmHg)	75.8 ± 10.3	75.9 ± 10.4	76.5 ± 10.4	77.7 ± 10.4	<0.01	a, b, c < d
Hypertension	545 (32.2)	569 (34.6)	748 (39.3)	1103 (53.7)	<0.01	a, b < c < d

Abbreviations: sUA, serum urate; WC, waist circumference; HC, hip circumference; BMI, body mass index; HDL-C, high-density lipoprotein cholesterol; lipoprotein cholesterol; GGT, gamma-glutamyl transferase; SBP, systolic blood pressure; DBP, diastolic blood pressure. * post-hoc analysis was conducted using Scheffe’s test, a: quartile 1, b: quartile 2, c: quartile 3, d: quartile 4.

**Table 2:** Anthropometric characteristics of premenopausal and postmenopausal women with hypertension.

Variables	Premenopausal Women (*N* = 1415)			Postmenopausal Women (*N* = 5879)		
	Without Hypertension (*N* = 1142)	With Hypertension (*N* = 273)	*p* *	Without Hypertension (*N* = 3187)	With Hypertension (*N* = 2692)	*p* *
sUA (mg/dL)	4.08 ± 0.87	4.42 ± 1.06	<0.01	4.35 ± 1.03	4.72 ± 1.25	<0.01
Age (year)	46.65 ± 3.98	48.57 ± 4.27	<0.01	63.08 ± 8.44	65.84 ± 7.82	<0.01
BMI (kg/m^2^)	24.10 ± 2.96	26.19 ± 3.55	<0.01	23.94 ± 3.15	25.06 ± 3.33	<0.01
WC (cm)	79.25 ± 8.18	84.26 ± 8.65	<0.01	82.35 ± 8.96	85.25 ± 8.98	<0.01
HC (cm)	93.45 ± 6.31	96.14 ± 6.39	<0.01	92.06 ± 6.48	93.54 ± 6.83	<0.01
SBP (mmHg)	111.00 ± 11.22	136.00 ± 16.44	<0.01	116.30 ± 12.00	136.90 ± 17.92	<0.01
DBP (mmHg)	72.43 ± 8.41	88.61 ± 9.81	<0.01	73.14 ± 7.97	81.14 ± 11.08	<0.01
TC (mg/dL)	190.40 ± 32.36	197.40 ± 33.90	0.002	205.48 ± 36.26	206.94 ± 37.99	0.14
TG (mg/dL)	114.80 ± 70.17	146.30 ± 94.17	<0.01	141.50 ± 78.67	162.50 ± 95.14	<0.01
HDL (mg/dL)	47.55 ± 11.10	46.49 ± 11.40	0.16	46.48 ± 11.10	44.55 ± 10.74	<0.01
Total protein (g/dL)	7.34 ± 0.38	7.43 ± 0.42	0.01	7.35 ± 0.40	7.42 ± 0.42	<0.01
Albumin (g/dL)	4.42 ± 0.22	4.44 ± 0.26	0.22	4.43 ± 0.23	4.48 ± 0.24	<0.01
GGT (I/U)	18.34 ± 27.48	22.56 ± 27.39	0.03	19.06 ± 20.14	22.38 ± 29.59	<0.01
Fasting glucose (mg/dL)	92.93 ± 15.41	98.30 ± 23.20	<0.01	97.10 ± 20.58	101.70 ± 23.00	<0.01
Creatinine (mg/dL)	0.85 ± 0.08	0.85 ± 0.11	0.88	0.86 ± 0.10	0.90 ± 0.25	<0.01
Current smoking (%)	7 (1.2)	2 (1.4)	0.93	90 (2.8)	66 (2.5)	0.53
Current drinker (%)	448 (39.2)	108 (39.6)	0.89	886 (27.8)	638 (23.7)	0.001
Regular exercise (%)	459 (40.2)	107 (39.2)	0.76	849 (26.6)	785 (29.2)	0.11

Abbreviations: sUA, serum urate; BMI, body mass index; WC, waist circumference; HC, hip circumference; SBP, systolic blood pressure; DBP, diastolic blood pressure; TC, total cholesterol; TG, triglyceride; HDL, high-density lipoprotein; GGT, gamma-glutamyl transferase. *, *p*-value was calculated by *t*-test.

**Table 3:** Effect of interactions between anthropometric parameters on hypertension in premenopausal and postmenopausal women.

Variables	Crude Odds Ratio (95% CI)		Adjusted Odds Ratio (95% CI)	
	Premenopausal (*N* = 1415)	Postmenopausal (*N* = 5879)	Premenopausal (*N* = 1415)	Postmenopausal (*N* = 5879)
Drinking status × sUA ^†^				
Never, past drinker and sUA < 5 mg/dL	1.00 (reference)	1.00 (reference)	1.00 (reference)	1.00 (reference)
Current drinker and sUA < 5 mg/dL	0.95 (0.69–1.30)	0.82 (0.71–0.95)	0.92 (0.60–1.42)	0.79 (0.66–0.95)
Never, past drinker and sUA ≥ 5 mg/dL	**2.16 (1.44–3.24)**	**1.96 (1.71–2.23)**	1.57 (0.88–2.77)	**1.91 (1.62–2.25)**
Current drinker and sUA ≥ 5 mg/dL	**2.66 (1.67–4.23)**	**1.42 (1.17–1.71)**	**2.09 (1.10–4.00)**	**1.44 (1.14–1.81)**
BMI × urate ^‡^				
Normal and sUA < 5 mg/dL	1.00 (reference)	1.00 (reference)	1.00 (reference)	1.00 (reference)
Obesity and sUA < 5 mg/dL	**3.11 (2.27–4.25)**	**1.85 (1.63–2.11)**	1.15 (0.69–1.92)	**1.61 (1.33–1.94)**
Normal and sUA ≥ 5 mg/dL	**2.32 (1.34–4.00)**	**1.82 (1.56–2.12)**	1.93 (0.92–4.06)	**1.86 (1.53–2.25)**
Obesity and sUA ≥ 5 mg/dL	**5.20 (3.48–7.78)**	**3.13 (2.67–3.66)**	**2.13 (1.14–3.99)**	**3.18 (2.54–3.96)**
Waist circumference × urate ^†^				
Normal and sUA < 5 mg/dL	1.00 (reference)	1.00 (reference)	1.00 (reference)	1.00 (reference)
Abdominal obesity and sUA < 5 mg/dL	**2.58 (1.87–3.56)**	**1.60 (1.41–1.82)**	0.77 (0.43–1.27)	0.86 (0.71–1.03)
Normal and sUA ≥ 5 mg/dL	**2.27 (1.46–3.53)**	**1.87 (1.59–2.18)**	**1.97 (1.08–3.60)**	**1.84 (1.51–2.24)**
Abdominal obesity and sUA ≥ 5 mg/dL	**4.59 (2.98–7.06)**	**2.74 (2.35–3.20)**	1.27 (0.64–2.52)	**1.62 (1.30–2.01)**
Triglyceride × sUA ^†^				
Normal and sUA < 5 mg/dL	1.00 (reference)	1.00 (reference)	1.00 (reference)	1.00 (reference)
Hypertriglyceridemia and sUA < 5 mg/dL	**2.03 (1.43–2.88)**	**1.50 (1.32–1.72)**	1.26 (0.78–2.06)	1.14 (0.96–1.34)
Normal and sUA ≥ 5 mg/dL	**2.19 (1.45–3.31)**	**1.78 (1.53–2.07)**	1.65 (0.92–2.96)	**1.82 (1.51–2.20)**
Hypertriglyceridemia and sUA ≥ 5 mg/dL	**3.88 (2.49–6.04)**	**2.63 (2.25–3.08)**	**2.38 (1.28–4.43)**	**2.08 (1.72–2.53)**

Abbreviations: sUA, serum urate; BMI, body mass index. † Adjusted for age, systolic blood pressure and BMI. ‡ Adjusted for age, systolic blood pressure and waist circumference. Statistically significant results were presented by bold type.

**Table 4:** Association of *XDH* genetic variants and risk of hypertension.

SNP		Crude	*p*	Model 1	*p*	Model 2	*p*
Number	Type						
rs206847	AA	1.000 (ref)		1.000 (ref)		1.000 (ref)	
	AC	2.467 (0.677–8.997)	0.17	2.519 (0.690–9.202)	0.16	3.174 (0.807–12.491)	0.1
	CC	3.631 (0.863–15.287)	0.08	3.755 (0.887–15.900)	0.07	5.076 (1.044–24.682)	**0.04**
rs206860	AA	1.000 (ref)		1.000 (ref)		1.000 (ref)	
	AG	0.287 (0.091–0.905)	0.03	0.282 (0.089–0.893)	0.03	0.263 (0.078–0.888)	**0.03**
	GG	0.661 (0.183–2.386)	0.53	0.646 (0.178–2.342)	0.51	0.451 (0.109–1.866)	0.27
rs207425	GG	1.000 (ref)		1.000 (ref)		1.000 (ref)	
	GA	2.065 (0.770–5.537)	0.15	2.044 (0.758–5.509)	0.16	3.160 (1.015–9.841)	**0.05**
	AA	-	0.99	-	0.99	-	0.99
rs1884725	GG	1.000 (ref)		1.000 (ref)		1.000 (ref)	
	GA	1.052 (0.338–3.279)	0.93	1.011 (0.323–3.162)	0.99	0.957 (0.282–3.244)	0.94
	AA	3.668 (0.805–16.725)	0.09	3.935 (0.854–18.127)	0.08	11.162 (1.525–81.706)	**0.02**
rs3769616	GG	1.000 (ref)		1.000 (ref)		1.000 (ref)	
	GA	-	0.99	-	0.99	-	0.99
	AA	1.580 (0.207–12.037)	0.66	1.562 (0.203–12.013)	0.67	9.732 (0.972–97.400)	0.05
rs206826	AA	1.000 (ref)		1.000 (ref)		1.000 (ref)	
	AC	0.351 (0.110–1.123)	0.08	0.355 (0.110–1.139)	0.08	0.282 (0.081–0.979)	**0.05**
	CC	0.907 (0.283–2.913)	0.87	0.905 (0.280–2.918)	0.87	0.588 (0.158–2.180)	0.43

Abbreviations: SNP, single nucleotide polymorphism; A, adenine, C, cytosine, G, guanine. Model 1 was adjusted for age, smoking status, alcohol consumption, regular exercise. Model 2 was adjusted for Model 1 and additionally adjusted for systolic blood pressure, total cholesterol and baseline body mass index. Statistically significant results were presented by bold type.

## Abbreviations

sUA: serum urate; BMI, body mass index; WC, waist circumference; HC, hip circumference; HDL-C, high density lipoprotein cholesterol; GGT, gamma-glutamyl transferase; SBP, systolic blood pressure; DBP, diastolic blood pressure.

## References

[1] Chang C.C., Wu C.H., Liu L.K., Chou R.H., Kuo C.S., Huang P.H., Chen L.K., Lin S.J. (2018). Association between serum uric acid and cardiovascular risk in nonhypertensive and nondiabetic individuals: The Taiwan I-Lan Longitudinal Aging Study. Sci. Rep..

[2] Orlando A., Cazzaniga E., Giussani M., Palestini P., Genovesi S. (2018). Hypertension in Children: Role of Obesity, Simple Carbohydrates, and Uric Acid. Front. Public Health.

[3] Krishnan E., Kwoh C.K., Schumacher H.R., Kuller L. (2007). Hyperuricemia and incidence of hypertension among men without metabolic syndrome. Hypertension (Dallas Tex. 1979).

[4] Jossa F., Farinaro E., Panico S., Krogh V., Celentano E., Galasso R., Mancini M., Trevisan M. (1994). Serum uric acid and hypertension: The Olivetti heart study. J. Hum. Hypertens..

[5] Chen Q., Yin Y.J., Chen W.Y., Wu J.N., Huang X. (2018). Assessment of the association between serum uric acid levels and the incidence of hypertension in nonmetabolic syndrome subjects: A prospective observational study. Medicine.

[6] Wei F., Sun N., Cai C., Feng S., Tian J., Shi W., Xu W., Wang Y., Yang X., Li W.D. (2016). Associations between serum uric acid and the incidence of hypertension: A Chinese senior dynamic cohort study. J. Transl. Med..

[7] Amos C.I., Wu X., Broderick P., Gorlov I.P., Gu J., Eisen T., Dong Q., Zhang Q., Gu X., Vijayakrishnan J. (2008). Genome-wide association scan of tag SNPs identifies a susceptibility locus for lung cancer at 15q25.1. Nat. Genet..

[8] Nieto F.J., Iribarren C., Gross M.D., Comstock G.W., Cutler R.G. (2000). Uric acid and serum antioxidant capacity: A reaction to atherosclerosis?. Atherosclerosis.

[9] Sciacqua A., Perticone M., Tassone E.J., Cimellaro A., Miceli S., Maio R., Sesti G., Perticone F. (2015). Uric acid is an independent predictor of cardiovascular events in post-menopausal women. Int. J. Cardiol..

[10] Cheng D., Du R., Wu X.Y., Lin L., Peng K., Ma L.N., Xu Y., Xu M., Chen Y.H., Bi Y.F. (2018). Serum Uric Acid is Associated with the Predicted Risk of Prevalent Cardiovascular Disease in a Community-dwelling Population without Diabetes. Biomed. Environ. Sci..

[11] Kawamoto R., Ninomiya D., Senzaki K., Kumagi T. (2018). Interaction between body mass index and serum uric acid in relation to blood pressure in community-dwelling Japanese men. Clin. Hypertens..

[12] Jin M., Yang F., Yang I., Yin Y., Luo J.J., Wang H., Yang X.F. (2012). Uric acid, hyperuricemia and vascular diseases. Front. Biosci. (Landmark Ed.).

[13] Corry D.B., Eslami P., Yamamoto K., Nyby M.D., Makino H., Tuck M.L. (2008). Uric acid stimulates vascular smooth muscle cell proliferation and oxidative stress via the vascular renin-angiotensin system. J. Hypertens..

[14] Kang D.H., Park S.K., Lee I.K., Johnson R.J. (2005). Uric acid-induced C-reactive protein expression: Implication on cell proliferation and nitric oxide production of human vascular cells. J. Am. Soc. Nephrol. JASN.

[15] Mazzali M., Hughes J., Kim Y.G., Jefferson J.A., Kang D.H., Gordon K.L., Lan H.Y., Kivlighn S., Johnson R.J. (2001). Elevated uric acid increases blood pressure in the rat by a novel crystal-independent mechanism. Hypertension (Dallas Tex. 1979).

[16] Wang J., Qin T., Chen J., Li Y., Wang L., Huang H., Li J. (2014). Hyperuricemia and risk of incident hypertension: A systematic review and meta-analysis of observational studies. PLoS ONE.

[17] Hak A.E., Choi H.K. (2008). Menopause, postmenopausal hormone use and serum uric acid levels in US women--the Third National Health and Nutrition Examination Survey. Arthritis Res. Ther..

[18] Sumino H., Ichikawa S., Kanda T., Nakamura T., Sakamaki T. (1999). Reduction of serum uric acid by hormone replacement therapy in postmenopausal women with hyperuricaemia. Lancet (Lond. Engl.).

[19] Scheepers L.E., Wei F.F., Stolarz-Skrzypek K., Malyutina S., Tikhonoff V., Thijs L., Salvi E., Barlassina C., Filipovsky J., Casiglia E. (2016). Xanthine oxidase gene variants and their association with blood pressure and incident hypertension: A population study. J. Hypertens..

[20] Battelli M.G., Bolognesi A., Polito L. (2014). Pathophysiology of circulating xanthine oxidoreductase: New emerging roles for a multi-tasking enzyme. Biochim. Biophys. Acta.

[21] Wu B., Hao Y., Shi J., Geng N., Li T., Chen Y., Sun Z., Zheng L., Li H., Li N. (2015). Association between xanthine dehydrogenase tag single nucleotide polymorphisms and essential hypertension. Mol. Med. Rep..

[22] Verdecchia P., Schillaci G., Reboldi G., Santeusanio F., Porcellati C., Brunetti P. (2000). Relation between serum uric acid and risk of cardiovascular disease in essential hypertension. The PIUMA study. Hypertension (Dallas Tex. 1979).

[23] Choi J.R., Ahn S.V., Kim J.Y., Koh S.B., Choi E.H., Lee G.Y., Jang Y.E. (2018). Comparison of various anthropometric indices for the identification of a predictor of incident hypertension: The ARIRANG study. J. Hum. Hypertens..

[24] Kim J., Yoon D.W., Lee S.K., Lee S., Choi K.M., Robert T.J., Shin C. (2017). Concurrent presence of inflammation and obstructive sleep apnea exacerbates the risk of metabolic syndrome: A KoGES 6-year follow-up study. Medicine.

[25] Choi J.R., Jang Y., Kim Yoon S., Park J.K., Sorn S.R., Park M.Y., Lee M. (2015). The Impact of CDH13 Polymorphism and Statin Administration on TG/HDL Ratio in Cardiovascular Patients. Yonsei Med. J..

[26] Bonora E., Capaldo B., Perin P.C., Del Prato S., De Mattia G., Frittitta L., Frontoni S., Leonetti F., Luzi L., Marchesini G. (2008). Hyperinsulinemia and insulin resistance are independently associated with plasma lipids, uric acid and blood pressure in non-diabetic subjects. The GISIR database. Nutr. Metab. Cardiovasc. Dis. NMCD.

[27] Ninomiya T., Kubo M., Doi Y., Yonemoto K., Tanizaki Y., Rahman M., Arima H., Tsuryuya K., Iida M., Kiyohara Y. (2007). Impact of metabolic syndrome on the development of cardiovascular disease in a general Japanese population: The Hisayama study. Stroke.

[28] Kanellis J., Kang D.H. (2005). Uric acid as a mediator of endothelial dysfunction, inflammation, and vascular disease. Semin. Nephrol..

[29] Xu J., Lloyd D.J., Hale C., Stanislaus S., Chen M., Sivits G., Vonderfecht S., Hecht R., Li Y.S., Lindberg R.A. (2009). Fibroblast growth factor 21 reverses hepatic steatosis, increases energy expenditure, and improves insulin sensitivity in diet-induced obese mice. Diabetes.

[30] Techatraisak K., Kongkaew T. (2017). The association of hyperuricemia and metabolic syndrome in Thai postmenopausal women. Climacteric J. Int. Menopause Soc..

[31] Johnson R.J., Kang D.H., Feig D., Kivlighn S., Kanellis J., Watanabe S., Tuttle K.R., Rodriguez-Iturbe B., Herrera-Acosta J., Mazzali M. (2003). Is there a pathogenetic role for uric acid in hypertension and cardiovascular and renal disease?. Hypertension (Dallas Tex. 1979).

[32] Roberts K.E., Kawut S.M., Krowka M.J., Brown R.S., Trotter J.F., Shah V., Peter I., Tighiouart H., Mitra N., Handorf E. (2010). Genetic risk factors for hepatopulmonary syndrome in patients with advanced liver disease. Gastroenterology.

[33] Li Y., Tang H., Qi H., Shen C., Sun L., Li J., Xu F., Jiao W., Yang X., Shen A. (2018). rs1800796 of the IL6 gene is associated with increased risk for anti-tuberculosis drug-induced hepatotoxicity in Chinese Han children. Tuberculosis.

[34] Yang J., Kamide K., Kokubo Y., Takiuchi S., Horio T., Matayoshi T., Yasuda H., Miwa Y., Yoshii M., Yoshihara F. (2008). Associations of hypertension and its complications with variations in the xanthine dehydrogenase gene. Hypertens. Res. Off. J. Jpn. Soc. Hypertens..

